# Rootstock Genotype Dictates Phosphorus Deficiency Tolerance and Transcriptional Plasticity in Grafted *Camellia oleifera* Plants

**DOI:** 10.3390/life15091489

**Published:** 2025-09-22

**Authors:** Zhihua Ren, Juan Liu, Jin Zeng, Li Cheng, Huiyun Liu, Yunyu Zhang, Qinhua Cheng, Wenjuan Su, Huaiyuan Wu, Dongnan Hu

**Affiliations:** 1 College Forestry, Jiangxi Agricultural University, Nanchang 330045, China; zhihuaren2023@163.com (Z.R.); suosuodecl@163.com (L.C.); huiyunliu@foxmail.com (H.L.); 2 Jiangxi Key Laboratory of Subtropical Forest Resources Cultivation, Nanchang 330045, China; 3 School of Mechanical Engineering, Wuhan Polytechnic University, Wuhan 420023, China; zengjin0819@163.com; 4 Xinyu Forestry Development Service Center, Xinyu 338099, China; yyzh1949@163.com (Y.Z.); ericwhy1993@163.com (H.W.); 5 Gannan Arboretum, Ganzhou 341299, China; qinhua19990315@163.com; 6 College of Forestry and Grassland, Nanjing Forestry University, Nanjing 210037, China; suwenj0126@163.com

**Keywords:** low-P stress, *Camellia oleifera*, rootstocks, transcriptomics

## Abstract

Rootstock choice offers a powerful lever for tailoring economically important trees to adverse environments. *Camellia oleifera* Abel., a premier oil-producing species cultivated widely on red-soil hills, suffers large yield losses under chronic phosphorus deficiency. We grafted a single elite scion (CL4) onto three contrasting rootstocks (CL4, CL3, CL53) and monitored growth and root transcriptomes for 1.5 years under adequate (1 mM) or limiting (0 mM) P supply. Under low-P stress, the rootstock identity reshaped the root architecture: CL4/CL3 produced the longest, most extensive network, increasing the total root length by 49.7%, the surface area by 52.9%, and the volume by 42.6% relative to the control, whereas leaf morphology responded solely to P supply, not to the graft combination. CL4/CL3 also accumulated up to more than 17.5% of root biomass and 28.25% of whole-plant biomass than any other combination. Physiologically, CL4/CL3 acted as an aggressive P miner, accumulating 67.8% more P in its roots than the self-grafted control under P limitation, while CL4/CL4 maximized the internal P use efficiency, showing a 44.74% higher root P use efficiency than CL4/CL53—two contrasting yet effective strategies for coping with low-P stress. Transcriptome profiling uncovered 1733 DEGs in the CL4/CL3 and 2585 in the CL4/CL4 roots, with 150 and 255 uniquely co-expressed genes, respectively. CL4/CL3 up-regulated organic-acid and phenylpropanoid pathways; CL4/CL4 activated defense and phosphate transport networks. qRT-PCR of six genes confirmed that CL4/CL3 mounted a stronger low-P response via MAPK, hormonal, and lipid–metabolic signaling. These results provide a mechanistic framework for rootstock-mediated P efficiency and establish a foundation for the molecular breeding of *C. oleifera* under nutrient-limited conditions.

## 1. Introduction

Phosphorus (P) is an essential element for plants, significantly influencing their growth and development [1]. It plays a vital role in various plant functions, such as metabolic regulation, energy transfer, and protein activation [2,3,4]. While P is mainly present in natural soil, it is mostly in the form of organophosphorus compounds, leading to a low concentration of inorganic phosphate (Pi) available for plant uptake [5]. Over the past century, the excessive use of P fertilizers has been driven by the need to meet food demands and maintain high crop yields but has resulted in increased costs and negative environmental impacts [6]. Moreover, the depletion of rock phosphate, a key component of these fertilizers, is a growing concern, with reserves projected to be exhausted within the next 50–100 years [7,8].

Native plant species have evolved unique strategies to adapt to low-Pi availability in soil [9]. Progress has been made in enhancing plant P acquisition efficiency through root foraging and phosphorus use efficiency (PUE) through P remobilization strategies [10,11]. For example, the presence of a growing number of root hairs and a relative high root-to-shoot ratio have been considered as symbiotic traits that contribute to high P acquisition efficiency [12,13]. Studies have shown that by increasing the length of the root system, the number of lateral roots, and the branching angle of the root system, plants increase the contact area between the root system and the soil, thereby increasing P absorption [11,12]. For instance, cotton plants respond to low-P stress by enhancing P absorption through increased lateral roots, branching density, and root hair length [14]. Furthermore, the response to Pi starvation is consistently regulated by gene regulatory networks, which involves numerous Pi transporters and Pi starvation-induced genes [15]. Many transcription factors, signal molecules, and other upstream regulators could be activated by Pi starvation. For example, the transcript factor WRKY has been demonstrated to participate in the regulation of Pi acquisition under low-Pi condition [16].

Grafting is a cornerstone horticultural technique that accelerates clonal propagation while preserving elite traits and stabilizing heterosis. By reshaping root architecture and physiology, it also enhances tolerance to environmental stresses, including water stress and nutrient deficiency [17,18]. Given that rootstocks are responsible for nutrient absorption, grafted plants can take up more ions and water compared to self-rooted plants and transport these to the scion. Utilizing the grafting technique to select a highly tolerant root system as a rootstock under nutrient deficiency can enhance nutrient absorption and/or utilization in plants. For example, Adam & Ulas (2023) reported that the tomato genotypes HelenaF1 and ALT exhibit exceptional rootstock potential, manifested through coordinated morphological, physiological, and biochemical traits that confer pronounced resilience under low-nitrogen conditions [19]. In citrus, the FA-5 rootstock confers superior drought tolerance by enhancing the osmotic adjustment capacity, thereby markedly improving the performance of grafted trees under water deficit conditions [20]. Yet, the rootstock-mediated mechanisms that orchestrate P acquisition, remobilization, and signaling under chronic P limitation—particularly in long-lived woody species—remain largely unexplored.

*Camellia oleifera* Abel. is an essential woody oil-producing tree species endemic to China. The tea oil extracted from the seeds of this species is highly valued for human health and is favored by consumers as a high-quality edible oil. With the growing oil demand, *C. oleifera* planting area has been continuously expanding in recent decades. It has been reported that the cultivated areas have reached 4.5 million ha in 2022 and will be 6 million ha in 2025. This species is naturally grown and widely planted in the acidic red soil regions of Southern China, which are characterized by a notable P deficiency. The shortage of Pi in these areas has significantly hampered the development of the *C. oleifera* industry [16]. In practice, the plants of *C. oleifera* are often propagated by sprout anvil grafting [21]. Many studies have focused on the variability in P among different scion varieties. For example, when the vigorous variety of the ‘Gan 8’ scion was grafted with the rootstock of ‘Ganwu 2’, it showed better performance in shoot P accumulation and root PUE under low-P stress [22]. A comprehensive assessment of seedling from twelve *C. oleifera* half-sib families revealed that the plants from ‘Changlin 4’ (CL4) half-sib families, which had higher root/shoot ratio, root area, and PUE, were categorized as P-efficient. On the other hand, the plants from ‘Changlin 3’ (CL3) half-sib families performed poorly in these features and were categorized as P-inefficient. However, the rootstock-mediated effect on low-P stress in *C. oleifera* has yet to be investigated. It is still unknown whether the tolerance of the grafted seedling to a low P supply would vary when different types of P-efficient plants are used as rootstocks for the same scion.

In this study, we investigated the response of grafted *C. oleifera* combinations, with the same commercial scion variety grafted onto three different rootstocks under P deficiency and control conditions (0 mM and 1 mM). The research focused on the following aspects: (1) the influence of rootstock and P availability on the morphology and biomass of the grafted plants; (2) the impact of rootstock and P availability on P accumulation and PUE of the grafted plants; (3) the effect of rootstock and P availability on unigenes and their underlying signaling pathways.

## 2. Materials and Methods

### 2.1. Plant Materials and Growth Conditions

The experiment was conducted at the Science Park of Jiangxi Agricultural University (115°49′ E, 28°45′ N), in Nanchang, Jiangxi Province, China. The locale is characterized by a subtropical monsoon climate, experiencing 147–157 precipitation days annually, with the annual rainfall ranging between 1600 and 1700 mm and an average annual temperature fluctuating between 17 °C and 17.7 °C. Building on our earlier findings [23], we employed three semi-sibling rootstocks that differ markedly in phosphorus use efficiency (PUE): CL3 (low PUE), CL4 (high PUE), and ‘Changlin 53’ (CL53; moderate–high PUE). The scion chosen for all grafting combinations was the ‘Changlin 4’ (CL4) cultivar. Consequently, we produced three grafted assemblies—CL4/CL3, CL4/CL4, and CL4/CL53 (scion/rootstock)—with the self-grafted CL4/CL4 acting as the internal benchmark, and CL4/CL3 and CL4/CL53 as the experimental grafts. This single-scion design stripped away scion variability and directly exposed each rootstock’s influence on phosphorus acquisition, echoing the commercial practice of pairing uniform elite clones with diverse rootstocks to tailor orchards to specific stresses.

In December 2018, *Camellia* seeds underwent cold stratification in sand, a process critical for germination. By May 2019, these germinated seeds were selected for anvil grafting. The grafting methodology was described by [22]. The germinated *C. oleifera* seeds were trimmed 2–3 cm above the seed, and the trimmed roots were extracted as rootstocks. Branches and leaves, selecting one leaf per scion, were collected from the *C. oleifera* germplasm resource nursery. The wedge-shaped tip of each scion branch was inserted into a V-shaped cleft in the root, which had been cut vertically for 1 cm, and was subsequently wrapped and then placed on the seedbed and cultivated to form a grafted plant for experimental treatment. A grafting substrate, a mixture of peat, perlite, and vermiculite in a 3:1:1 ratio (available P: 14.95 ± 0.26 mg/kg, available potassium: 464.39 ± 3.34 mg/kg, hydrolyzable nitrogen: 419.01 ± 6.77 mg/kg), was utilized, resulting in a high survival rate among all grafted plants, without significant variance in compatibility among the three graft combinations. In January 2021, a critical transplantation stage was initiated, transferring the plants into pots with a river sand substrate (available P: 1.06 ± 0.14 mg/kg, available potassium: 27.19 ± 0.33 mg/kg, hydrolyzable nitrogen: 5.36 ± 1.23 mg/kg). Commencing in June 2020, healthy and uniform grafted plants were subjected to experimental treatments. Prior trials showed that *C. oleifera* survives in P-impoverished red soils; 0 mM P has therefore been our validated threshold for P-stress studies. Therefore, P regimes were set as 1 mM (control) and 0 mM (deficiency). KH_2_PO_4_ was used as the P source. Since June 2020, the grafted plants were irrigated monthly with 100 mL of 1/2 Hoagland nutrient solution (no P included). Standard horticultural practice was maintained by applying 100 mL of tap water weekly and adjusting the amount according to weather, increasing irrigation during hot periods and reducing it when it rained. To disentangle acute from chronic responses to phosphorus limitation, we implemented a staggered-harvest time series (0, 30, 90, and 180 d). In November 2021, all grafted plants were collected and immediately dissected into distinct tissues for downstream analyses. Each treatment combination was represented by three independent biological replicates to guarantee the robustness and reproducibility of our findings.

### 2.2. Determination of the Morphology

Leaf morphometrics, including leaf area, length, width, and perimeter, were quantitatively assessed using a specialized leaf area measuring instrument of the handheld laser leaf area meter CI-203-CID (CID Bio-Science, Camas, WA, USA). Subsequently, the leaf shape index was calculated as the ratio of leaf length to leaf width. Three measurement rounds were taken on every plant (*n* = 3 per treatment, all plants measured, no random selection).

The entire root systems of the *C. oleifera* grafted plants were carefully excavated and cleansed for root analysis. The roots were then meticulously severed at the branching nodes and arranged in a transparent plastic container for imaging. Utilizing a scanner Expression 10000XL3.49 (Epson America, Los Alamitos, CA, USA), high-resolution scans of the entire root system were obtained. Advanced image analysis was performed using the Win Rhizo Pro2012b (Regent Instruments Inc., Quebec, QC, USA) software, enabling the precise quantification of total root length, surface area, and volume.

### 2.3. Determination of Biomass and P Concentration

All grafted plants were meticulously harvested. The roots and shoots were segregated and individually encased in brown paper envelopes. At each of the four harvest time points, triplicate samples were taken from three different grafted plants per treatment and initially placed in an oven set at 105 °C for two hours, followed by a drying phase at 65 °C until a constant weight was achieved. Each tissue’s mass was precisely measured using an electronic balance with a sensitivity of 0.01 g. The root-to-shoot ratio and total dry weight were subsequently calculated. The root-to-shoot ratio, expressed in g/g, was determined as the root weight divided by the shoot weight. The total dry weight, in grams, was computed as the sum of the dry weights of the root and shoot.

For P content analysis, three replicated samples per treatment were analyzed using the molybdenum–antimony colorimetric method, with measurements conducted via a GENESYS 180 UV-Vis spectrophotometer (Thermo Fisher Scientific, Waltham, MA, USA). Phosphorus use efficiency (PUE) was estimated based on the total P accumulation by the plant. P accumulation, measured in mg, was calculated as the product of P concentration (mg/g) and dry weight (g). PUE, denoted in g/mg, was determined as the dry weight divided by the total P accumulation.

### 2.4. RNA Extraction, Illumina Sequencing, and Data Analysis

In order to elucidate the P deficiency rootstock-mediated adaptability in *C. oleifera*, we focused on the rootstocks CL3 and CL4 because of their differential performance under the P deficiency condition and further explored the underlying molecular mechanisms using root transcriptome sequencing. In this study, the roots of the CL4/CL3 (scion/rootstock) combination in the control and P deficiency treatment groups were designated as R34P1 and R34P0, respectively. Similarly, the roots of the CL4/CL4 combination under control and low-P conditions were labeled as R44P1 and R44P0, respectively. Each treatment had three replicates.

Total root RNA extraction was performed using TRIzol^®^ reagent (Thermo Fisher Scientific, Waltham, MA, USA) according to the manufacturer’s protocol. RNA integrity and quantity were assessed using a 5300 Bioanalyzer Agilent (Agilent Technologies, Santa Clara, CA, USA) and an ND-2000 spectrophotometer (Thermo Fisher Scientific, Waltham, MA, USA), respectively. RNA samples with high quality were selected for the construction of the sequencing library.

The RNA-seq transcriptome library was prepared using Illumina^®^ Stranded mRNA Prep and a ligation method, starting with 1 μg of total RNA. Subsequently, double-stranded cDNA synthesis was performed using the SuperScript double-stranded cDNA synthesis kit (Thermo Fisher Scientific, Waltham, MA, USA) with random hexamer primers from Illumina. Post-quantification with Qubit 4.0 (Thermo Fisher Scientific, Waltham, MA, USA), the paired-end RNA-seq sequencing library was processed on the NovaSeq 6000 sequencer (Illumina, San Diego, CA, USA), generating reads of 2 × 150 bp. RNA purification, reverse transcription, library construction, and sequencing were completed by Shanghai Majorbio Bio-pharm Biotechnology Co., Ltd., Shanghai, China.

In this study, raw paired-end reads were meticulously trimmed and subjected to quality control using fastp with its default parameters. Clean reads were independently aligned to the reference genome in orientation mode utilizing HISAT2 (http://ccb.jhu.edu/software/hisat2/index.shtml, accessed on 5 November 2022). Subsequently, StringTie was employed in a reference-based approach to assemble the mapped reads of each sample. Differentially Expressed Genes (DEGs) between two distinct samples were identified by calculating the expression level of each transcript using the Transcripts Per Million reads (TPM) method, quantifying gene abundances. Further, functional enrichment analysis, including Gene Ontology (GO) and Kyoto Encyclopedia of Genes and Genomes (KEGG) pathways, identified DEGs significantly enriched in GO terms and metabolic pathways, with a Bonferroni-corrected *p*-value of ≤0.05, in comparison to the entire transcriptome background. GO functional enrichment and KEGG pathway analyses were executed.

### 2.5. Quantitative Real-Time PCR Analysis

Several candidate genes were selected to validate the transcript expression using quantitative real-time PCR (qRT-PCR). RNA extraction was carried out using RNAprep Pure DP441 (Tiangen, Beijing, China). The reverse transcription of RNA to synthesize cDNA was performed according to the iScript^TM^ cDNA Synthesis Kit protocol (Bio-Rad, Hercules, CA, USA), utilizing a PCR instrument 846-x-070-301 (Bio-Rad, Hercules, CA, USA) set to a specific temperature and time program (25 °C for 5 min, 46 °C for 20 min, 95 °C for 1 min, and holding at 4 °C).

Primers for these genes were designed based on the sequencing results, using the online tool available at Primer3 (https://www.primer3plus.com/, accessed on 12 October 2023) online and synthesized by Shanghai Majorbio Bio-pharm Biotechnology Co., Ltd., Shanghai, China (Table 1). EF1a1 was chosen as the internal reference gene. Real-time quantitative detection was conducted using iTaq^TM^ Universal SYBR^®^ Green Supermix 1725121 (Bio-Rad, Hercules, CA, USA) in a CFX Connect^TM^ Real-Time System 788BR07708 (Thermo Fisher Scientific, Waltham, MA, USA), following a temperature and time program of 95 °C for 2 min, 95 °C for 5 s, 60 °C for 30 s, for 40 cycles, with a final to obtain a melt curve (95 °C for 5 s, 65 °C for 5 s, and 95 °C for 5 s). The relative gene expression levels were quantified using the 2^−∆∆Ct^ method.

### 2.6. Statistical Analysis

Two-factor analysis of variance (ANOVA) was employed to investigate the effects of rootstock varieties and P availability, as well as their interaction effects, in the different grafted plants. One-way ANOVA was employed to assess differences between the control and the P deficiency conditions using Student’s *t*-test at the 5% significance level.

## 3. Results

### 3.1. Effects of Different Rootstocks on the Morphology of C. oleifera Grafted Plants

**Figure 1:**
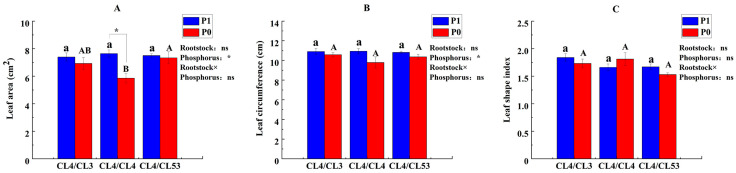
Effects of the rootstock on leaf morphology of the grafted plants under control and low-P conditions. (**A**) Leaf area, (**B**) leaf circumference, and (**C**) leaf shape index. Lowercase and uppercase letters on the bars indicate significant differences in grafting combinations between control and P deficiency supply treatments at the *p* ≤ 0.05 level, respectively. (The statistical test used was one-way analysis of variance). The upper right of the figure shows the ‘Interaction Effect Analysis’ (the statistical test used was two-way analysis of variance); ‘Rootstock’, ‘Phosphorus’, and ‘Rootstock × Phosphorus’ indicate ‘the impact of rootstock genotypes’, ‘the impact of phosphorus’, and ‘the interaction of rootstock genotypes and phosphorus’, respectively. ‘*’ indicate that the interactions are significant at the *p* ≤ 0.05 level, respectively, and ‘ns’ indicate no significant difference.

In this study, the morphological characters of leaves and roots were assessed among the *C. oleifera* plants grafted with CL4, CL3, and CL53 rootstocks under control and P deficiency conditions. The results showed that P availability could affect significantly the leaf area and leaf circumference of the *C. oleifera* grafted plants (Figure 1), but no significant difference in leaf morphologies was found among grafted plants with different rootstock genotypes.

**Figure 2:**
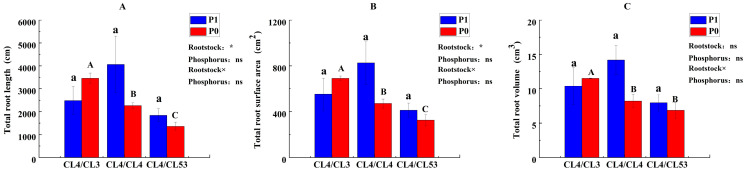
Root morphology of *C. oleifera* plants. (**A**) Total root length, (**B**) total root surface area, and (**C**) total root volume. Lowercase and uppercase letters on the bars indicate significant differences in grafting combinations between control and P deficiency supply treatments at the *p* ≤ 0.05 level, respectively. (The statistical test used was one-way analysis of variance). The upper right of the figure shows the ‘Interaction Effect Analysis’ (the statistical test used was two-way analysis of variance); ‘Rootstock’, ‘Phosphorus’, and ‘Rootstock × Phosphorus’ indicate ‘the impact of rootstock genotypes’, ‘the impact of phosphorus’, and ‘the interaction of rootstock genotypes and phosphorus’, respectively. ‘*’ indicate that the interactions are significant at the *p* ≤ 0.05 level, respectively, and ‘ns’ indicate no significant difference.

The results of the root morphology analysis indicated that P availability did not affect the root characters. The rootstock genotypes significantly affected the root length and total root surface area but did not influence the total root volume (Figure 2). Among the *C. oleifera* plants grafted with different rootstocks, CL4/CL3 boosted the total root length by 49.7%, the surface area by 52.9%, and the root volume by 42.6% relative to the control under P deficiency.

### 3.2. Effect of the Rootstock on the Biomass of Grafted C. oleifera

**Figure 3:**
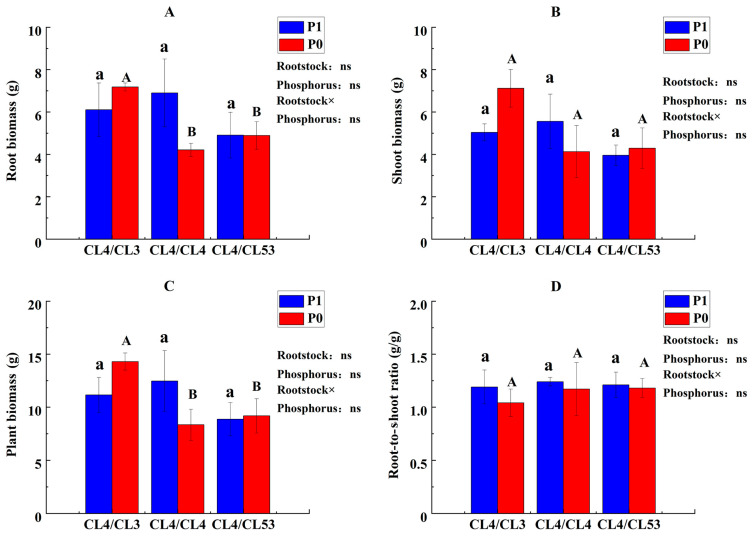
Biomass of each part of the grafted plants. (**A**) Root biomass, (**B**) shoot biomass, (**C**) whole-plant biomass, and (**D**) root/shoot ratio. Lowercase and uppercase letters on the bars indicate significant differences in grafting combinations between control and P deficiency supply treatments at the *p* ≤ 0.05 level, respectively. (The statistical test used was one-way analysis of variance). The upper right of the figure shows the ‘Interaction Effect Analysis’ (the statistical test used was two-way analysis of variance); ‘Rootstock’, ‘Phosphorus’, and ‘Rootstock × Phosphorus’ indicate ‘the impact of rootstock genotypes’, ‘the impact of phosphorus’, and ‘the interaction of rootstock genotypes and phosphorus’, respectively. ‘ns’ indicate no significant difference.

The results of the biomass of *C. oleifera* indicated that root biomass, shoot biomass, whole-plant biomass, and root/shoot ratio in the grafted plants of three rootstocks were not affected by P availability and the rootstock genotypes (Figure 3). However, we found that different grafted plants had different performance under the P deficiency treatment. For example, the root biomass and the entire plant biomass of CL4/CL3 were much greater compared to those of the other grafted plants. Under P deficiency treatment, the root biomass of CL4/CL3 increased by 17.51% compared with that of the control and was 4.06% higher compared to that of CL4/CL4. The whole-plant biomass of CL4/CL3 under low P supply was improved by up to 28.25% compared to that of the control.

### 3.3. Effects of the Rootstock on P Content, P Accumulation, and PUE in the Grafted C. oleifera

**Figure 4:**
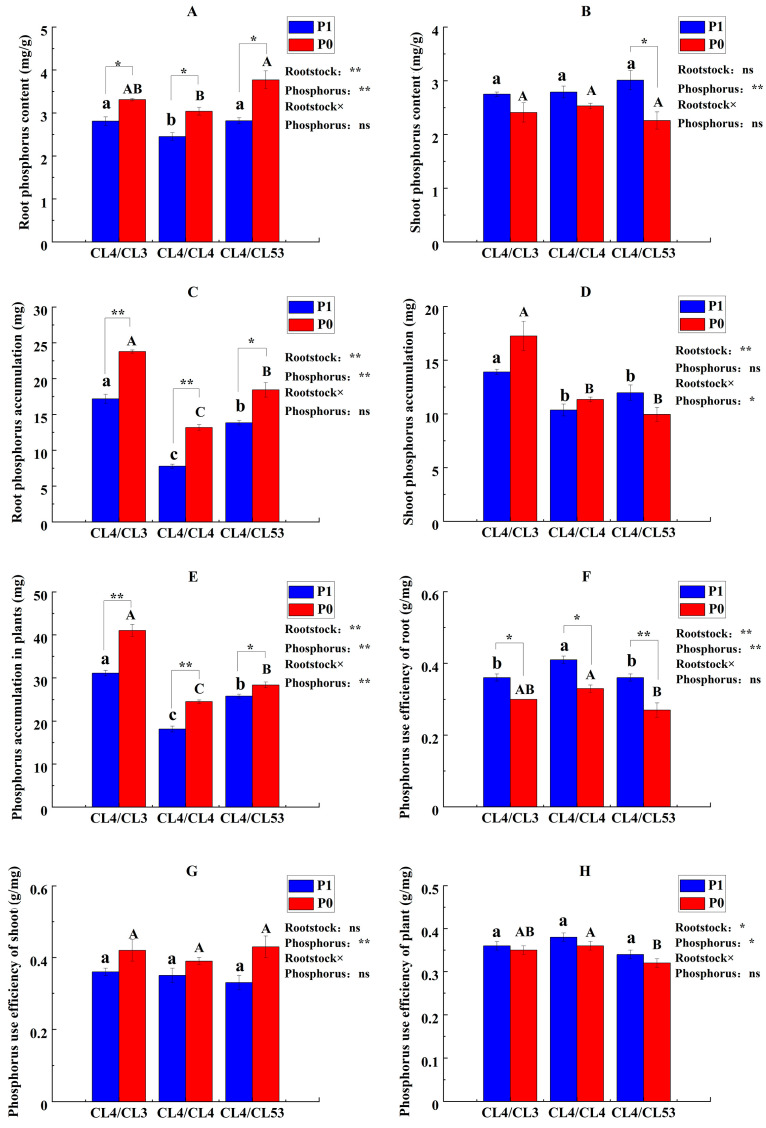
P content, P accumulation, and PUE in each part of the grafted plants. (**A**) Root P content, (**B**) shoot P content, (**C**) root P accumulation, (**D**) shoot P accumulation, (**E**) whole-plant P accumulation, (**F**) root PUE, (**G**) shoot PUE, and (**H**) plant PUE. Lowercase and uppercase letters on the bars indicate significant differences in grafting combinations between control and P deficiency supply treatments at the *p* ≤ 0.05 level, respectively. (The statistical test used was one-way analysis of variance). ‘*’ and ‘**’ on the bars indicate a significant difference between control and low-P-treated plants at the *p* ≤ 0.05 and *p* ≤ 0.01 levels, respectively. (The statistical test used was Student’s test). The upper right of the figure shows the ‘Interaction Effect Analysis’ (the statistical test used was two-way analysis of variance); ‘Rootstock’, ‘Phosphorus’, and ‘Rootstock × Phosphorus’ indicate ‘the impact of rootstock genotypes’, ‘the impact of phosphorus’, and ‘the interaction of rootstock genotypes and phosphorus’, respectively. ‘*’ and ‘**’ indicate that the interactions are significant at the *p* ≤ 0.05 and *p* ≤ 0.01 levels, respectively, and ‘ns’ indicate no significant difference.

We further investigated the P content, P accumulation, and PUE among the *C. oleifera* plants grafted with three rootstock genotypes in different tissues under control and P deficiency availability. The findings indicated that root P content, root P accumulation, and root PUE in the grafted plants were significantly affected by both rootstock genotypes and P availability (Figure 4). Meanwhile, whole-plant P accumulation and whole-plant PUE exhibited similar performance and were significantly affected by both rootstock×P, rootstock variety, and P availability. But shoot P content, shoot accumulation, and shoot PUE in the grafted plants were mainly affected by P availability.

Among the three graft combinations, CL4/CL3 achieved the greatest P accumulation in roots, shoots, and whole plants under P-limited conditions; for instance, root P accumulation was 67.8% higher than in the self-grafted CL4/CL4 control. On the other hand, CL4/CL4 exhibited modestly superior root P use efficiency—44.74% higher than that of CL4/CL53—and, under P deficiency, maintained the highest whole-plant PUE while accumulating less P in every tissue under both treatments. It seemed that CL4/CL3 and CL4/CL4 had different strategies to adapt to a P-deficient environment. The former showed high P-mining capacity by increasing root P absorption from the soil and accumulating P in the whole plant to cope with the P deficiency. The latter enhanced P use efficiency by optimizing the P application rate to meet the plant’s P demand. Therefore, CL4/CL3 and CL4/CL4 were selected for further transcriptomic analysis under two P availability levels in this study to clarify the underlying mechanisms.

### 3.4. Transcriptomic Analysis for Different Grafted Plants Under Two Treatments

**Figure 5:**
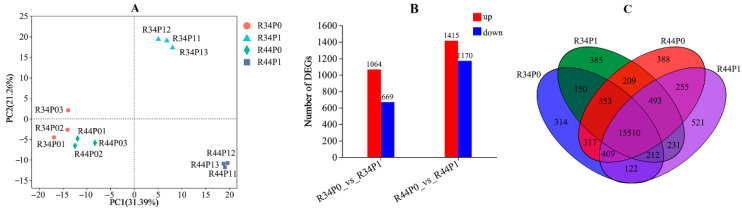
Comparative analysis of differential genes under control and low P conditions. (**A**) PCA analysis of the samples, (**B**) up-regulated and down-regulated DEGs, and (**C**) Venn diagram with the number of DEGs. ‘R34P01’ indicates ‘R + 3 + 4 + P0 + 1’, which represents ‘root + rootstock (CL3 or CL4) + scion (CL4) + phosphorus level (P0 or P1) + repeat (1, 2 or 3)’.

**Figure 6:**
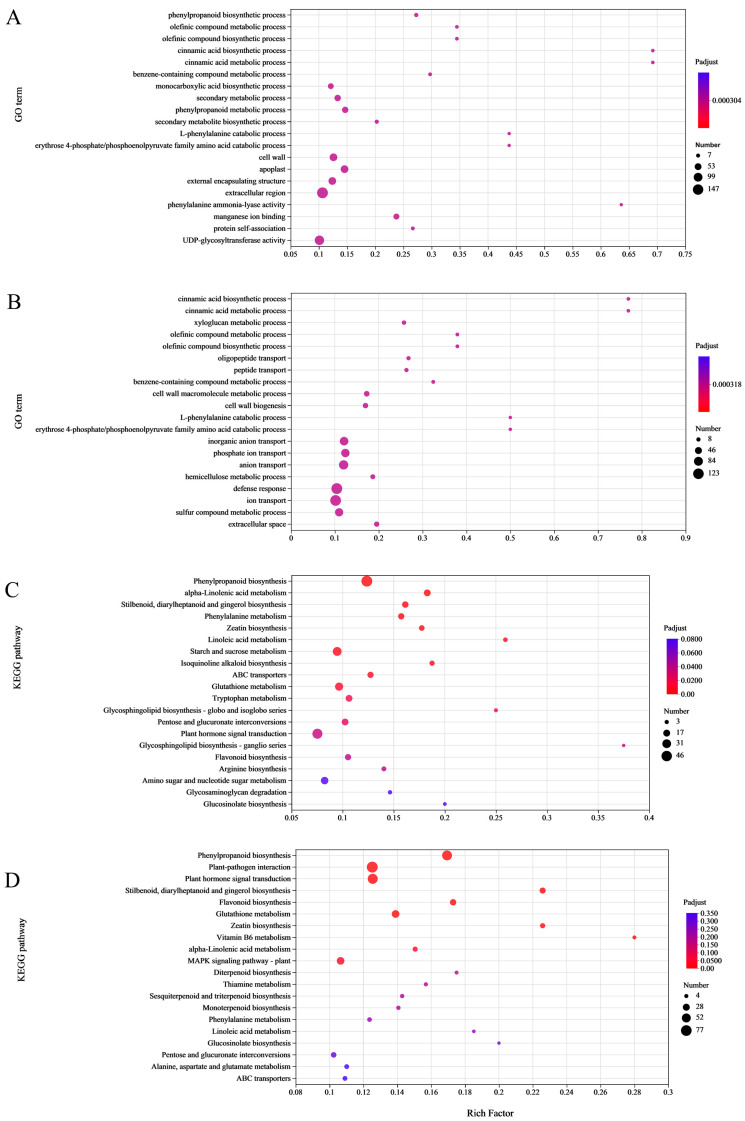
GO and KEGG enrichment analysis. (**A**) R34P1 vs. R34P0 GO enrichment analysis of DEGs, (**B**) R44P1 vs. R44P0 GO enrichment analysis of DEGs, (**C**) R34P1 vs. R34P0 KEGG enrichment analysis of DEGs, and (**D**) R44P1 vs. R44P0 KEGG enrichment analysis of DEGs.

Based on the different performance on P content, P accumulation, and PUE under low P levels, the roots of the CL4/CL3 (scion/rootstock) and CL4/CL4 grafted combinations were chosen for further transcriptional sequencing analysis to investigate the response mechanisms of plants grafted with different rootstocks (Figure 5 and Figure 6). PCA analysis of all samples revealed a strong correlation within each treatment and significant differences between the treatment groups, indicating sufficient reliability for further analysis (Figure 5A). The results of DGE analysis between control and P deficiency conditions showed that there were 1064 up-regulated genes and 669 down-regulated genes in the CL4/CL3 roots, while 1415 up-regulated and 1170 down-regulated genes were found in the CL4/CL4 roots (Figure 5B). The Venn diagram was used to present the presence of uniquely expressed genes in each of the four combinations. The results indicated that there were 150 co-expressed genes in the CL4/CL3 combination under the two different treatments, and 255 genes in the CL4/CL4 combination (Figure 5C).

The GO enrichment analysis revealed distinct functional specializations of root genes in the CL4/CL3 and CL4/CL4 graft combinations when investigating the DEGs of each combination under the two P levels (Figure 6). When compared to the DEGs of the CL4/CL3 roots under control and P deficiency conditions, gene functions primarily related to monocarboxylic acid biosynthesis, phenylpropane metabolism, secondary metabolite biosynthesis, phosphoenolpyruvate family amino acid catabolism, and cinnamic acid metabolism (Figure 6A). The DEGs in the CL4/CL4 roots under the two different treatments were primarily involved in pathways associated with defense reaction, hemicellulose metabolism, phosphate ion transport, phosphoenolpyruvate family amino acid catabolism, and cell wall macromolecular metabolism (Figure 6B).

KEGG analysis based on the DEGs in the CL4/CL3 roots under the two P levels showed a significant enrichment in phenylalanine metabolism, starch and sucrose metabolism, glutathione metabolism, pentose and glucuronate interconversions, plant hormone signal transduction, glycosphingolipid biosynthesis, and flavonoid biosynthesis. KEGG analysis of the CL4/CL4 roots under the two different treatments indicated a notably enrichment in phenylpropane biosynthesis, plant–pathogen interaction, plant hormone signaling, flavonoid biosynthesis, glutathione metabolism, zeatin biosynthesis, MAPK signaling pathway, and other metabolic pathways.

**Figure 7:**
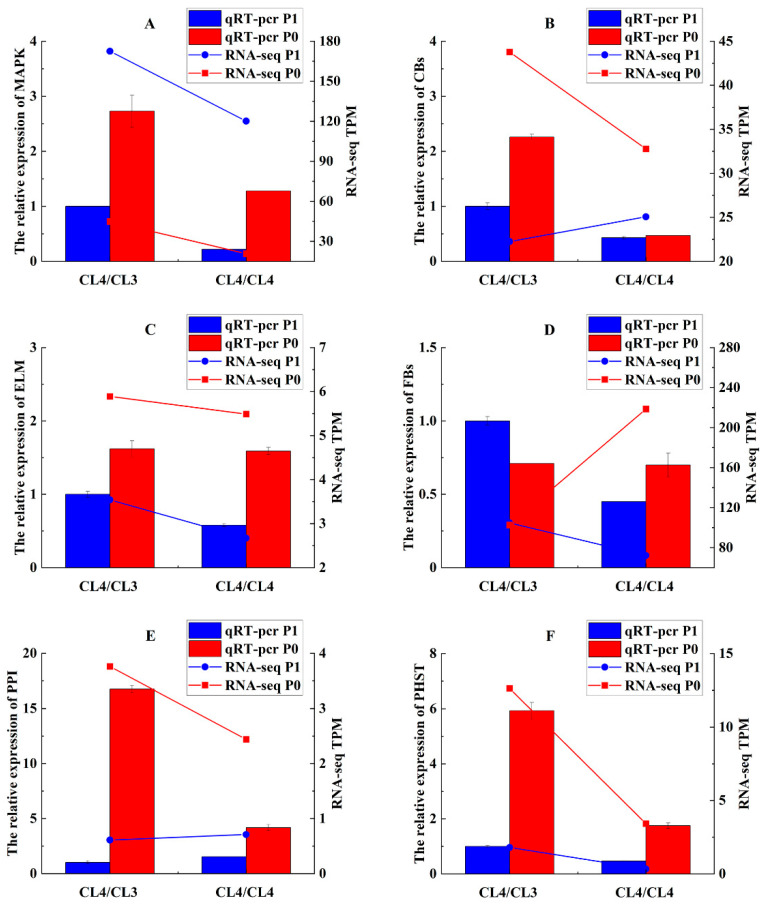
Real-time quantitative expression analysis and RNA-Seq validation analysis. (**A**) MAPK, (**B**) CBs, (**C**) ELM, (**D**) FBs, (**E**) PPI, and (**F**) PHST.

In order to verify the accuracy of the gene expression analysis, six genes based on the transcriptomic sequencing analysis were selected to conduct qRT-PCR under the two P treatments (Table 1). The results showed that the relative gene expression using RNA-Seq and qRT-PCR of five genes were matched (except for MAPK), indicating that the transcriptomic sequencing analysis was reliable (Figure 7). For example, the expression of MAPK was induced in the CL4/CL3 grafted combination under P deficiency, with an expression level 2.73 times higher than that in the control. In the CL4/CL4 grafted combination, the expression level of MAPK was 1.28 times higher than that in the control. The gene *CBs* in the grafted CL4/CL4 showed similar low expression levels under both treatments. But the expression level of this gene in CL4/CL3 under P deficiency was 2.26 times higher than that in the control. The lipid metabolism genes *ELM* and *FBs* are involved in P metabolism. The expression of *ELM* gene was induced by P deficiency, with similar performance in both grafted combinations. The expression of the *FBs* gene was repressed in the two grafted plants under P deficiency conditions compared to the control. The expression of PPI (a gene involved in plant–pathogen interaction) in both grafted plants was significantly induced in P deficiency conditions. And the expression level of this gene in CL4/CL3 was much higher than in CL4/CL4. The expression of PHST (a plant hormone signaling gene) was also induced under P deficiency conditions, being three times higher in CL4/CL3 than in CL4/CL4. This suggests that the P deficiency condition could induce the expression of hormone-related genes, Overall, P deficiency conditions stimulate intracellular signaling, leading to the secretion of hormones and metabolites like carotenoids, which then regulate lipid metabolism and facilitate the plant’s response to P deficiency.

## 4. Discussion

Breeding highly nutrient-efficient varieties has been demonstrated as an effective way to address the nutrient shortage [24]. Grafting scion varieties maintains good bud characteristics while improving the plant’s ability to adapt to environmental stress [25,26]. A growing body of research has begun to dissect how rootstocks modulate plant performance under phosphorus-limited conditions, with particular attention given to apple [27], peach [28], and grapevine [29]. In this study, we examined the effects of P deficiency on the same scion of *C. oleifera* grafted with different rootstocks. The results showed that the rootstocks of the *C. oleifera* grafted plants did not affect the leaf morphology but had a significant impact on the root length and total root surface area. Moreover, different grafted plants were found to have different root biomass, root P content, and PUE under P deficiency. The CL4/CL3 grafted combination performed better in nutrient absorption under P-limited conditions.

Phosphorus deficiency has been widely documented to impair growth and development across a broad range of crop species, including soybean [30] and cotton [31,32]. In this study, under P-limited conditions, the leaf area and leaf circumference were reduced apparently in three grafted combinations. This is because P deficiency could reduce stomatal opening, with less captured CO_2_ and reduced triose phosphate, significantly influencing the recycling of NADPH and ATP, thus inhibiting plants’ photosynthetic capacity and growth [33]. Moreover, rootstocks have been reported to alter scion morphology in some fruit trees, including apple [34,35], peach [36], and pear [37], where dwarfing or invigorating rootstocks can modulate leaf size, thickness, and specific leaf area via changes in endogenous hormone levels and hydraulic conductance. In this study, the three examined rootstock genotypes had little impact on the leaf morphologies of the grafted plants. Rootstocks would be more likely to modulate below-ground traits than above-ground leaf architecture under P limitation. In this study we observed that the three rootstocks scarcely altered scion leaf morphology, yet significantly changed root length and surface area. This divergence suggests that rootstock-mediated P acquisition operates primarily through root plasticity—enhancing soil exploration and P uptake—rather than through alterations in leaf size or shape.

Grafting is often considered to improve the nutrient uptake of plants. In this study, the rootstocks significantly affected the root morphology, which is supported by the findings of other studies [38,39]. Liu et al. [40] demonstrated that apple scions grafted onto *Malus prunifolia* or Chistock #1 produced significantly longer, more vigorous root systems than their non-grafted counterparts. Under the low P level condition, the rootstock CL3 performed better than the rootstock CL4. Although the latter showed a little higher evidence of changes in root morphology, there was no significant differences between the two rootstocks under the P-sufficient condition. Furthermore, the rootstock CL3 seemed to also have greater P accumulation and P content in the roots. Leaf functional traits allow for plant survival and maintain plants’ ecosystem function [41]. Through grafting, the rootstock has the capability to modify the phenotype of certain scions. The leaf area was affected positively by the rootstock in mango [42]. Specifically, soybean rootstocks can influence the chlorophyll levels in the leaves, thereby impacting the process of leaf aging [43]. Consequently, rootstocks that align with desired traits are often chosen for grafting in agricultural production. For chili peppers, employing drought-resistant rootstocks can shield the plants from oxidative stress due to water scarcity, enhancing their drought tolerance [44]. Different apple rootstock types uniquely influence pollen germination, with specific varieties enhancing cold stress tolerance during flowering [45]. In this study, it was found that the rootstock did not have a significant impact on leaf morphology. However, the rootstocks did have a significant effect on P accumulation in the shoot. This could be attributed to the interaction between the rootstocks and the scions. The influence of the rootstock–scion interaction has been observed and discussed in various crops and fruit trees, including soybeans [30], cotton [31], and apple [34,35,40]. Some researchers have proposed that the apparent interaction between rootstocks and scions for certain traits in the grafted plants is primarily controlled by the rootstocks. For example, potato grafting using late blight-resistant varieties as rootstocks could enhance resistance to late blight in scions derived from susceptible varieties by up-regulating the expression of disease-resistant genes in the scions [41]. The contents of sugars, acids, and volatiles in heterografted tomato pericarps are influenced by the rootstocks [46]. It was further demonstrated that rootstocks play an essential role in grafting. Our results further demonstrated that grafting the scion CL4 to the rootstock CL3 improved the plant growth and increased the P content more than grafting to the rootstocks CL4 and CL53 under low-P stress, which suggests that rootstock may have a crucial regulating effect on grafted *C. oleifera*.

The mechanisms underlying the response to low-P conditions in *C. oleifera* have been widely discussed by many researchers. Su et al. [16] suggested that *CoWRKYs* in the P-efficient CL40 variety of *C. oleifera* may play a crucial role in P deficiency tolerance, being involved in the transportation and recycling of P in the leaves and affecting diverse metabolic pathways. Three hub target genes, *ARF22*, *WRKY53*, and *SCL6*, which may play key roles in CL166 of *C. oleifera* in controlling transcriptomic regulation in response to low Pi stress, were identified [47]. In this study, some pathways were identified related to low P levels in oil-tea species, which were overlooked by previous studies. For example, mitogen-activated protein kinase (MAPK) signaling pathways play a very important role in plant abiotic stress [48]. Under low-P stress, *MAPK* was strongly up-regulated. It significantly modified plant growth under stress conditions and dramatically modified the root architecture through transcriptional regulation of the auxin transport-associated genes [49]. Overexpression of a member of the *MAPK* gene family enhanced tobacco’s dry mass production, Pi uptake capacity, and root system architecture establishment under low-Pi stress [50]. It is important to note that our study did not find differences in the expression of transcription factors common in the low-P response. For example, Su et al. [16] indicated that 32 *CoWRKYs* participated in the regulation of responses to P deficiency stress in *C. oleifera*. The complexity of the rootstocks–scion interaction-dominated P response mechanism may account for this phenomenon.

Moreover, this study delivers an integrated, molecular-level roadmap illustrating how distinct rootstocks steer the low-phosphorus adaptive strategies of grafted *C. oleifera*. GO and KEGG profiling exposed sharp functional partitioning between the two graft combinations, with each rootstock–scion pair deploying a unique transcriptional program to confront P limitation. CL4/CL3 preferentially up-regulated phenylpropanoid–flavonoid metabolism, organic-acid biosynthesis, glutathione-mediated antioxidant systems, and phytohormone signaling, thereby constructing a metabolic network centered on efficient P acquisition, mobilization, and translocation. In contrast, CL4/CL4 prioritized defense responses, cell wall remodeling, and *MAPK* cascades, reflecting a ‘defend-and-conserve’ strategy that enhanced P use efficiency rather than P uptake. This functional separation aligned with phenotypic data: CL4/CL3 exhibited marked increases in root length and surface area and a 28% rise in whole-plant P accumulation under low P, whereas CL4/CL4 improved the PUE while maintaining the biomass. The *MAPK* signaling cascade was the most strikingly differentially expressed pathway. qRT-PCR confirmed that *MAPK* expression in the CL4/CL3 roots reached 2.73-fold that of the control—significantly higher than the 1.28-fold increase in CL4/CL4—indicating that *MAPK*-mediated auxin transport and root architectural remodeling underpin the superior P acquisition of CL4/CL3. Similarly, the stronger up-regulation of the hormone-related gene *PHST* and the defense gene PPI in CL4/CL3 not only intensified the immediate root response to P limitation but may also have activated systemic acquired resistance, indirectly reducing P diverted to defense. Moreover, the up-regulation of the lipid metabolism gene *ELM* and the down-regulation of *FBs* jointly suggest coordinated membrane lipid turnover and P recycling. Collectively, rootstock–scion interactions do not merely sum additive effects; instead, they reprogram the root transcriptional networks to confer either a ‘CL3-type P-capture’ or a ‘CL4-type P-saving’ strategy under low P.

## 5. Conclusions

In this study CL4 scions were grafted onto three rootstocks and exposed to low P or control conditions. CL3 markedly improved scion P uptake and P use efficiency (PUE); under low P it stimulated its own root growth, which translated into greater biomass and total P in the shoot. Transcript profiling further suggested that CL3 primed the scion by up-regulating MAPK, hormone, and defense-related genes, although these candidates still await direct validation. Together the data underline the pivotal role of rootstock choice in grafted crops grown on P-deficient soils and provide a molecular framework for future rootstock–scion studies. The next steps are to confirm that the CL3 low-P advantage persists in the field and to test its performance under drought and in combinations with other commercial scion varieties.

## Figures and Tables

**Table 1:** Primer Information.

Name	Gene_id	Description	Primer Sequences
*MAPK*	augustus_masked-HiC_scaffold_ 13-processed-gene-1462.3	MAPK signaling pathway	F: CACTCGCCACAAACAATCCC
			R: TACGCTTGTGGGTTGTCGAA
*CBs*	snap_masked-HiC_scaffold_ 6-processed-gene-823.4	Carotenoid biosynthesis	F: CTCAGAGCGTGATGGGGATC
			R: ATTCTTGTTGAGCCGAGGCA
*ELM*	maker-HiC_scaffold_ 14-snap-gene-732.64	Ether lipid metabolism	F: TACCCATGGTGGTTGCCTTC
			R: ACGGGCCTGACTAATTGCAT
*FBs*	augustus_masked-HiC_scaffold _10-processed-gene-1746.20	Fatty acid biosynthesis	F: GTCCCACTCCGACATTCTCC
			R: TGGTTAAGTCGGTTGGAGGC
*PPI*	augustus_masked-HiC_scaffold _1-processed-gene-131.29	Plant–pathogen interaction	F: CCAGTGGCGGAGTCCAAATA
			R: GGGCGGGTCATGATGTAGAG
*PHST*	snap_masked-HiC_scaffold _14-processed-gene-167.29	Plant hormone signal transduction	F: GTGCCCTCTCAAATGGTGGA
			R: GGCCTAAGCTAGCACTACCG

## Data Availability

The transcriptomic data presented in this study are deposited in NCBI, accession number PRJNA1328430.
